# Combined nerve and tendon transfer (CNaTT) for grasp and release function in patients with tetraplegia: a matched prospective pilot study

**DOI:** 10.1177/17531934251381202

**Published:** 2025-11-24

**Authors:** Lina Bunketorp Käll, Therese Ramström, Johan Berg, Hannes Granberg, Carina Reinholdt, Johanna Wangdell

**Affiliations:** 1 Department of Occupational Therapy and Physiotherapy, Sahlgrenska University Hospital/Mölndal, Sweden; 2 Department of Health and Rehabilitation, Institute of Neuroscience and Physiology, Sahlgrenska Academy, Gothenburg University, Sweden; 3 Department of Hand Surgery, Centre for Advanced Reconstruction of Extremities, Sahlgrenska University Hospital/Mölndal, Sweden; 4 Institute of Clinical Sciences, Department of Hand Surgery, Sahlgrenska Academy, Gothenburg University, Sweden

**Keywords:** Combined nerve and tendon transfer, grasping, spinal cord injury, supinator branch to the posterior interosseous nerve (S-PIN) transfer, tetraplegia, upper limb

## Abstract

**Introduction:**

Traditional single-stage tendon transfer procedures in patients with tetraplegia are designed to restore active pinch and grasp function. However, digital extension is usually achieved by wrist tenodesis. More recently, nerve transfer has allowed potential restoration of digital extension. This matched observational pilot study compares clinical outcomes after traditional tendon transfers vs. a study group using two-stage using combined nerve and tendon transfer.

**Methods:**

Eighteen patients in the traditional tendon transfer control group and 18 patients in the combined nerve and tendon transfer group using a supinator branches to posterior interosseous nerve (S-PIN) transfer were compared. Primary outcomes include hand opening capacity, using the cylinder test. Secondary outcomes include muscle strength, first web space opening, grip and pinch strength, grasp and release ability, and activity performance.

**Results:**

Analyses indicated that adding a nerve transfer allowed more patients to achieve normal one-handed hand opening, although this difference was not statistically significant. Secondary outcomes revealed superior grasp and release ability and pinch opening in the study group; however, there were no notable differences in grip strength and activity performance between the groups.

**Conclusions:**

Adding a S-PIN nerve transfer appears to enhance grasp function in the tetraplegic limb when combined with established tendon transfer techniques. For individuals with tetraplegia, the capacity to use single hand grips is particularly significant, as it allows the other arm to stabilize and compensate for limited core balance. More participants with a longer follow-up are needed to fully demonstrate the superiority of combined nerve and tendon procedures.

**Level of evidence:**

III

## Introduction

Reconstructive surgery of the upper limb has emerged as a viable option for enhancing the function of paralysed upper extremities in patients with tetraplegia. Traditional tendon transfer and more recently, nerve transfer procedures have both been shown to effectively restore motion, balance, and hand function (Berg et al., 2024; Bunketorp-Kall et al., 2017a; 2017b; Fridén and Gohritz, 2015; Hill and Fox, 2019; Javeed et al., 2022; van Zyl et al., 2019; Wangdell et al., 2014).

Traditional single-stage tendon transfer procedures in individuals with tetraplegia are designed to restore active pinch and grasp function (Fridén et al., 2011), which ideally requires both digital flexion and extension. However, owing to limited availability of donor muscles with sufficient strength, these tendon transfer techniques cannot always reliably restore both active finger and thumb extension. Fridén et al. (2011) previously described various tenodesis procedures in combination with the tendon transfers which somewhat facilitates active opening of the first web space, and thereby enhance independence in daily activities (Wangdell et al., 2013). However, hand opening remains somewhat passive and restricted, despite reliable restoration of pinch and grasp (Berg et al., 2024; Fridén et al., 2011).

To expand reconstructive options for individuals with tetraplegia, various nerve transfer techniques have evolved over the past decade (Cain et al., 2015; Fridén and Gohritz, 2015; van Zyl et al., 2019). This represents a significant advance in the treatment of spinal cord injuries, increasing the number of patients eligible for surgery (Wilson, 2019). Bertelli et al. (2010) presented a successful case report of the transfer of supinator branches to the posterior interosseous nerve (S-PIN), enabling reanimation of thumb and finger extensors, and allowing for the potential restoration of independent extension and radial abduction of the thumb through reinnervation of the abductor pollicis longus (APL) muscle, and wrist extension resulting from reinnervation of the extensor carpi ulnaris (ECU). Despite the remarkable heterogeneity across studies on nerve transfers in tetraplegia, early results are promising (Fox et al., 2015). Nerve transfer can be effectively combined with tendon transfers in appropriately selected patients (Dibble et al., 2021), including those with mid-to-high-level cervical injuries (Berg et al., 2024). Combining the S-PIN procedure with tendon transfer techniques offers a unique opportunity to restore both active opening and strong closing of the hand (Berg et al., 2024; Fridén and Lieber, 2019). This integrated approach, referred to as combined nerve and tendon transfer (CNaTT), has the potential to maximize functional gains compared with either technique used alone.

Many unresolved questions remain about the advantages and disadvantages of performing nerve and tendon transfer procedures alone or in combination (Käll et al., 2021; van Zyl et al., 2019). To better assess the value of combining these approaches in individuals with tetraplegia, we have previously emphasized the importance of carefully matching control subjects on key variables (Berg et al., 2024). Building on this, we designed a new cohort study with a new control group, matched by injury level, where the study group was expanded, and the range of measurement tools broadened. The aim was to compare two groups, the first of which underwent traditional reconstruction of grasp using tendon transfer and the second that received CNaTT. The hypothesis is that a combined procedure may allow for better grasp and release in these patients, ultimately enhancing overall hand function in this patient population.

## Methods

### Study design and participants

This matched prospective observational controlled pilot study aims to establish preliminary data for this population. The study group consists of patients who underwent CNaTT performed as a two-stage procedure. The control group includes patients who previously underwent traditional tendon transfer procedures. The main outcome for the CNaTT group was assessed a minimum of 1 year after the tendon transfer procedure. Eligibility criteria for patients in the CNaTT group included: no other medical or neurological disorders; spinal cord injury with the International Standards for Neurological Classification of Spinal Cord Injury (ISNCSCI) motor level C5–C7; ⩾ Medical Research Council (MRC) (Compston, 2010) 4 strength in the muscle supplied by the donor nerve (supinator), with finger extensor strength ⩽ MRC 1; medical stability to undergo surgery as determined by a physician; minimal spasticity (Ashworth 1) in the forearm muscles; MRC 4 strength in the brachioradialis and wrist extension (if used as donors in reconstruction of grasp); maximum age of 55; and time since injury ⩽ 12 months. The controls were matched based on (1) type and number of active tendon transfers, (2) injury level according to the International Classification for Surgery of the Hand in Tetraplegia (ICSHT) and (3) sex. This pilot study serves as the first step in a larger research protocol designed to inform the planning and modification of the main study, which has been approved by the Ethical Review Authority (Dnr 2019-05838). All patients provided written informed consent, and the study was conducted in accordance with the Declaration of Helsinki.

### Surgical procedures

#### Nerve transfer

The patients in the study group underwent the first surgical procedure (nerve transfer) unilaterally or bilaterally. In this procedure, supinator motor nerves were transferred to the S-PIN as described by Bertelli et al. (2010). The surgery was performed in combination with unilateral triceps reconstruction via tendon transfer (posterior deltoid to triceps with tibialis anterior graft), where appropriate. After surgery, the sutured nerve must not be stretched. In a few early cases, elbow extension may be limited by 30°, and a hinged brace may be used for 2 weeks to prevent full extension, depending on perioperative findings. Following a brief postoperative rest period of 2 weeks, patients were instructed to strengthen the nerve transfer by activating the donor muscle (Kahn and Moore, 2016), but no other therapy related to the nerve transfer surgery was provided. Muscle strength following the S-PIN nerve transfer was monitored for at least 12 months. The outcomes of this evaluation guided the planning and customization of the second-stage tendon transfer procedure, including decisions on thumb stabilization and intrinsic balancing.

#### Tendon transfer for hand closing and balancing of the hand

The active tendon transfers typically included the brachioradialis to flexor pollicis longus (BR–FPL) transfer and extensor carpi radialis longus to flexor digitorum profundus (ECRL–FDP) transfer (Fridén et al., 2011). In the CNaTT group, the balancing procedures varied depending on the muscle strength (MRC) of the target muscles following the nerve transfer procedure. A typical ICSHT 4 patient with some finger extension underwent a split flexor pollicis longus to extensor pollicis longus (FPL–EPL) tenodesis, along with static or simplified intrinsic balancing procedures – passive reconstruction of the interossei according to House (McCarthy et al., 1997) and carpometacarpal joint capsulodesis. If the EPL was not sufficiently strong (less than MRC grade 4) to counterbalance the BR–FPL transfer, an EPL tenodesis was performed. Additionally, depending on the strength of the extensor digiti minimi (EDM) (minimum MRC grade 3), a third tendon transfer was performed by suturing the EDM to the abductor pollicis brevis (APB) for active thumb abduction (EDM–APB). In cases of weaker EDM function, a tenodesis was performed instead to assist with thumb positioning. Our specific early active rehabilitation protocol was initiated within 24 h postoperatively and has been previously described in detail (Wangdell et al., 2016).

### Outcome measures

#### Hand opening and grasp ability

**Figure 1.**
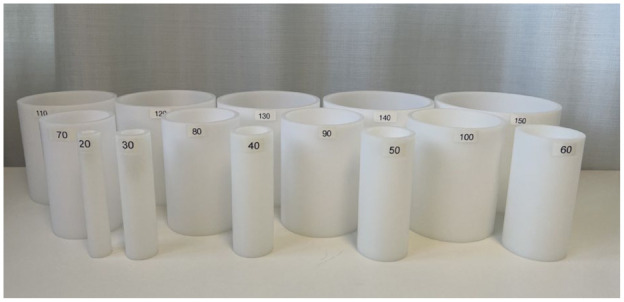
The cylinder test battery, composed of 14 stackable hard-plastic cylinders with diameters ranging from 20 to 150 mm.

The primary outcome measure was grasp ability, assessed using the cylinder test. This test is currently undergoing a reliability assessment, with test–retest reliability evaluated in the present study. The cylinder test measures the ability to perform an active or passive cylinder grasp in various forms, including normal, adapted, self-assisted, or examiner-assisted, as outlined in the instruction manual. The assessment battery consists of 14 plastic cylinders of varying diameters, ranging from 20 to 150 mm (Figure 1). Each subtest is scored separately from 20 to 150, reflecting different grasping techniques, with higher scores indicating a larger hand opening.

The strength of the extensor digitorum communis (EDC) was reported as the median score for all four digits (MRC grading) with the wrist and the MCP joints positioned at 0° (neutral position). The opening of the first web space was measured by the maximum active distance between the fingertip of the index finger and the thumb, with the wrist in the patient’s preferred position, using a plain ruler to measure in centimetres. Since the first web space measurement does not differentiate between radial and palmar abduction of the thumb, we also included *functional* pinch opening, measured by the maximum active distance between the proximal interphalangeal (PIP) joint of the index finger and the tip of the thumb, again with the wrist in the preferred position. To quantify the *functional* opening of the pinch grasp, an additional sub-category was added to the cylinder test, measuring the maximum width of the pinch grasp in millimeters.

#### Hand closing and grip strength

Grip strength was measured using a Jamar hydraulic hand dynamometer (JAMAR^®^ 5030J1, Sammons Preston Rolyan, USA), while pinch strength was assessed with a Preston Pinch Gauge (European Bissel Healthcare Ltd, Winchester, UK) with a 22 mm width. Both measurements were conducted according to the standardized position guidelines established by the American Society of Hand Therapists (Mathiowetz et al., 1985). The maximum score from three attempts was recorded for each measurement.

Grasp and activity performance were measured using the grasp and release test (GRT) (Reinholdt and Fridén, 2013; Wuolle et al., 1994). The GRT is a pick-and-place test that requires participants to unilaterally acquire, move, and release six objects of varying sizes and weights. The total number of successful repetitions for all six objects was recorded during a 30 s trial. For the blocks, only grasping using the pinch grip was accepted.

Activity was assessed using the Tetraplegia Upper Limb Activity Questionnaire (TUAQ) (Wangdell et al., 2022a, 2022b). The TUAQ consists of two separate unidimensional scales that evaluate performance and satisfaction in daily activities reliant on upper extremity function for individuals with tetraplegia. Each scale includes the same 10 items, with a five-point response option ranging from 1 to 5. Items are summed to produce a raw score for each scale, which is then converted to interval scores. The TUAQ serves as both a clinical assessment tool and an outcome measure following interventions targeting upper limb function in tetraplegia. Previous studies have demonstrated good psychometric properties, including reliability and agreement for individuals with tetraplegia, as well as responsiveness and construct validity for surgical reconstruction of hand function in this population (Wangdell et al., 2022b). Self-perceived hand function was rated using a visual analogue scale ranging from 0 to 10 (de Boer et al., 2004).

### Statistical analyses

Demographics and clinical characteristics were summarized using descriptive statistics. Comparisons between groups were conducted using analysis of covariance, adjusting for matching variables (injury level, active tendon transfers and sex) as well as predictors such as years from injury to surgery and years from surgery to follow-up. All tests were two-tailed and conducted at a significance level of *p* < 0.05. A graphical assessment of the normality assumption was performed through visual inspection of quintile–quintile plots of the model residuals. Test–retest reliability of the cylinder test was evaluated using the quadratic weighted kappa, interpreted as follows: values ⩽ 0 indicate no agreement; 0.01–0.20 indicate no to slight agreement; 0.21–0.40 indicate fair agreement; 0.41–0.60 indicate moderate agreement; 0.61–0.80 indicate substantial agreement; and 0.81–1.00 indicate almost perfect agreement (McHugh, 2012). All analyses were performed by using SPSS v.22.0 (IBM Corp., Armonk, NY, USA).

## Results

The demographics and clinical characteristics of the study cohort are presented in Table 1. There were 18 patients in the traditional tendon transfer control group and 18 patients in the CNaTT study group. The distribution of women and men was equal in both groups.

**Table 1.** Demographics and clinical characteristics of study participants.

Demographics	Study group (*n* = 18)	Control group (*n* = 18)	SMD
Gender			
Male	12	12 (67)	
Female	6	6	
Age at follow-up	38.4 (11.4)	42.6 (10.0)	0.647
Age at the time of the first surgery (years, mean, SD)	33.7 (11.3)	32.4 (9.0)	0.041
Time elapsed since last surgery (years, mean, SD)	4.2 (1.3)	10.6 (3.9)	2.780
Operated arm			
Right	9	9	
Left	9	9	
Had undergone triceps reconstruction	4	5	
Strength (MRC grade) in triceps (median, range)	4 (1–5)	4 (0–5)	
Cause of injury			
Transport activities	8	9	
Sport and leisure activities	10	7	
Falls	0	2	
ICSHT			
2	6	7	
3	7	5	
4	3	5	
5	2	1	
Level of injury (ISNCS)*			
C5	5	6	
C6	9	8	
C7	4	4	

Data are presented as numbers and percentages unless otherwise noted. SMD = Standardized mean difference; MRC = Medical Research Council.

* Descriptions of motor groups according to the International Classification for Surgery (ICSHT) of the Hand in Tetraplegia.

### Outcome of S-PIN transfer

The strength of the reanimated muscles in the study group, resulting from the S-PIN procedure, is presented in Table 2. In the study group, 10/18 patients achieved a median MRC grade of 3 or above in the reanimated EDC, while 13/18 patients reached an MRC grade of 3 or above in EPL. Ten patients exhibited some degree of reanimation in the ECU, although only three achieved MRC ⩾ 3.

**Table 2.** Strength (MRC grade) of the reanimated muscles in the study group, as a result of the supinator to posterior interosseus nerve (S-PIN) transfer.

MRC	ECU	EDC	EPL	APL
0	8	1	1	12
1	4	3	3	3
2	3	2	1	1
3	0	2	3	2
4	2	9	9	0
5	1	1	1	0

APL = Abductor pollicis longus; ECU = extensor carpi ulnaris; EDC = extensor digitorum communis; EPL = extensor pollicis longus; MRC = Medical Research Council.

Table 3 presents a summary of all procedures included in the traditional surgical approach for restoring grasp. There was no difference between groups with respect to the matching criteria in terms of the number of active tendon transfers. The majority of patients in both groups underwent two active tendon transfers for hand closure, utilizing BR or ECRL as donor tendons. The remaining patients had one active tendon transfer. Extensor digiti minimi transferred to APB transfer was perfomed in half of the patients in the study group to facilitate active palmar abduction and opposition of the thumb, but was not possible in the control group.

**Table 3.** Details of the surgical procedures in the study groups.

Surgical procedures	Study group	Control group
	Upper limbs = 18	Upper limbs = 18
*Tendon transfers*		
BR to FPL	14	14
ECRL to FDP	11	11
BR to FDP	4	4
EDM to APB	9	N/A
*Stabilization of CMC1*		
Capsulodesis	15	1
CMC1 arthrodesis	0	17
*Intrinsic reconstruction*		
House procedure	17	14
Zancolli Lasso procedure	1	1
*Balancing procedures*		
Split FPL–EPL tenodesis	14	10
ELK–extensor pollicis longus loop-knot	0	6
Tenodesis FPL – radius (passive pinch)	5	4
ECU tenodesis	5	11
EPL–EPB tenodesis	8	8
FDP tenodesis	1	0

APB = Abductor pollicis brevis; BR = brachioradialis; CMC1 = first carpometacarpal joint; ECRL = extensor carpi radialis longus; ECU = extensor carpi ulnaris; EDM = Extensor digiti minimi; ; EPB = extensor pollicis brevis; FDP = flexor digitorum profundus; FPL = flexor pollicis longus.

**Figure 2.**
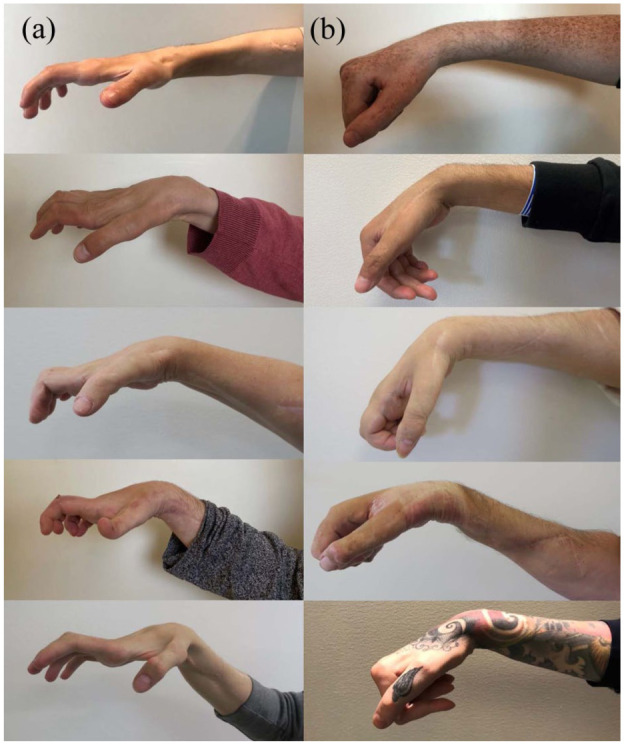
Right hand appearance at follow-up during an attempt at maximum hand opening. The combined nerve and tendon transfer (CNaTT) group (A, left column) shows greater wrist, finger and thumb extension compared with the control group (B, right column).

**Figure 3.**
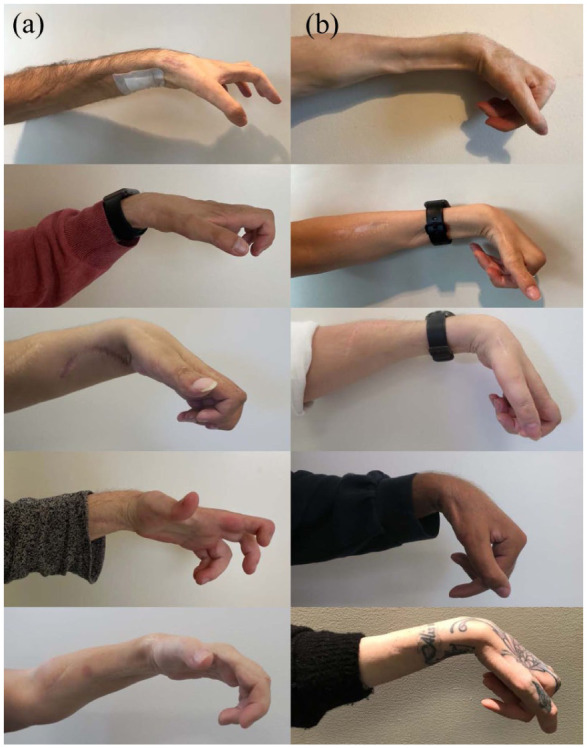
Left hand appearance at follow-up during an attempt at maximum hand opening. The combined nerve and tendon transfer (CNaTT) group (A, left column) demonstrates more pronounced wrist, finger and thumb extension compared with the control group (B, right column).@

Findings for the primary and secondary outcome measures are summarized in Table 4. Analyses using ANCOVA, indicated a tendency toward somewhat larger normal one-handed hand opening in the study group as measured by the cylinder test (16.7 mm, SD = 27.2) compared with the controls (0.0 mm, SD = 0.0), yet not reaching the statistical significance threshold (mean difference = 21.3 mm, 95% CI [−1.5 to 44.1]; *p* = 0.07). Furthermore, the study group exhibited significantly larger pinch opening, as measured by the cylinder-pinch subtest (34.4 mm, SD = 25.0), compared with the controls (10.0 mm, SD = 15.1) (mean difference = 32.8 mm, 95% CI [7.5 to 58.2]; *p* = 0.01). There were no significant differences between groups regarding the other components of the cylinder test. The functional pinch grasp was significantly larger in the study group (5.2 cm, SD = 2.4) as compared with controls (1.9 cm, SD = 1.6); *p* = 0.02, whereas the first webspace opening was 6.1 cm (SD = 3.1) in the study group and 3.9 cm (SD = 1.7) in the control group, with no significant difference between groups (*p* = 0.21). Figures 2 and 3 illustrate the appearance of the right and left hands, respectively, during the follow-up assessment when participants in the CNaTT group (A, left column) and control group (B, right column) aimed to achieve maximum hand opening.

**Table 4.** Primary and secondary outcomes and group comparisons.

Variable	Study group (*n* = 18)	Control group (*n* = 18)	Mean difference (95% CI)
*Cylinder test*			
One-handed – Normal	16.7 (27.2) 0.0 (0–80)	0.0 (0.0) 0.0 (0–0)	21.3 (−1.5 to 44.1) *p =* 0.07
One-handed – Adapted	36.1 (30.3) 30.0 (0–90)	16.1 (16.9) 15.0 (0–50)	24.2 (−6.0 to 54.5) *p =* 0.11
Two-handed	54.4 (31.1) 55.0 (0–90)	35.0 (27.1) 35.0 (0–70)	12.3 (−21.9 to 46.6) *p =* 0.47
Placing	88.9 (27.0) 90.0 (40–130)	67.2 (32.9) 70.0 (20–150)	21.1 (−17.5 to 59.8) *p =* 0.27
JAMAR max	5.1 (2.6) 4.3 (1.7–9.5)	4.6 (2.9) 4.5 (0.0–9.8)	0.0 (−2.8 to 2.9) *p =* 0.97
PINCH GAUGE max	1.8 (0.9) 1.5 (0.5–3.8)	1.5 (0.8) 1.5 (0.6–3.5)	0.8 (−0.3 to 1.8) *p =* 0.16
*Pinch opening*			
First webspace opening	6.1 (3.1) 6.0 (0.0–11.0)	3.9 (1.7) 4.0 (0.5–6.0)	1.8 (−1.1 to 4.7) *p =* 0.21
Functional pinch-grasp*	5.2 (2.4) 5.3 (0.5–8.5)	1.9 (1.6) 1.3 (0.0–5.5)	3.0 (0.5 to 5.5) ***p =* 0.02**
Cylinder test – PINCH	34.4 (25.0) 35.0 (0–80)	10.0 (15.1) 5.0 (0–50)	32.8 (7.5 to 58.2) ***p =* 0.01**
GRT total	119.3 (55.7) 107.5 (21–223)	73.9 (30.3) 68.0 (35–147)	47.2 (2.8 to 91.6) ***p =* 0.04**
TUAQ performance	67.3 (8.5) 53 ( 36–88)	51.8 (3.0) 55 (32–64)	15.5 (−4.7 to 35.8) *p =* 0.23
TUAQ satisfaction	50.4 (6.6) 41.5 (28–72)	40.4 (2.3) 43 (27–52)	9.9 (−5.7 to 25.5) *p =* 0.31

Descriptive data are presented as mean (standard deviation) and median (range). Statistical analyses were performed using analysis of covariance adjusted for matching variables (number of active tendon transfers and ICSHT), sex, years from injury to grip reconstruction and years from surgery to follow-up. Abbreviations: CI = confidence interval; GRT = grasp and release test; TUAQ = Tetraplegia Upper Limb Activity Questionnaire.

* Measured by the maximum active distance between the PIP joint of the index finger and the thumb with the wrist in preferred position, measured in centimetres

The total score for the GRT differed significantly between the groups; the study group outperformed the control group in their ability to pick up, move, and release six objects of varying sizes, weights, and textures using a palmar or lateral grasp. There were no significant differences between groups regarding whole hand grip strength (JAMAR) and pinch strength (PINCH GAUGE). Activity performance measured by the TUAQ did not differ significantly between the groups. The calculation of agreement for the test-retest reliability of the primary outcome measure indicated a Kappa value of 0.45 for one-handed cylinder grip (95% CI [0.09 to 0.72]), suggesting moderate test agreement.

## Discussion

In this study, we investigated the outcomes of traditional tendon transfer vs. CNaTT in matched study participants based on key variables relevant to reconstructive hand surgery in tetraplegia. Adjusted for matching variables and covariates, the results indicated a trend towards a significantly larger one-handed cylinder grasp in the study group compared with the controls. The CNaTT group also demonstrated a significantly larger functional pinch grip opening and cylinder pinch opening. Additionally, the study group demonstrated significantly improved abilities to acquire, move, and release objects of varying sizes and weights according to the GRT. All these achievements necessitate a certain degree of hand opening to effectively grasp and manipulate specific objects and the potential advantage of including a S-PIN transfer to future reconstructive algorithms. The CNaTT group, benefiting from reinnervated extensor muscles, was able to initiate active opening, which probably facilitated improved object manipulation, as evidenced by their superior performance in the GRT and pinch opening assessments. This enhanced ability to grasp and release objects, as demonstrated by the GRT, can probably be attributed to the restored capacity for hand and pinch opening in preparation for grasping.

Releasing objects through active extension requires relatively little strength, whereas achieving a power grip through active flexion demands significantly more power. The combination of nerve and tendon transfers therefore appears to offer an optimal approach for functional reconstruction of grasp. By having more active extension, the digits are able to go through a larger range of motion during flexion, thus influencing the speed of grasping, as suggested by the GRT results. The improved grasp speed together with the ability to perform one-handed grasping, as shown in the cylinder test, has the potential to facilitate a faster and smoother daily life by enabling smooth bilateral activities with the hands. For individuals with tetraplegia, the capacity to use single hand grips is particularly significant, as it allows the other arm to stabilize and compensate for limited core balance. This dual functionality can potentially expand the overall workspace, enabling one hand to reach and grasp while the other arm maintains stability.

There were no significant differences in grip strength between the groups. This is unsurprising as both groups underwent the same type of tendon transfers aimed at restoring active pinch and/or cylinder grip. However, it could be argued that enhanced hand opening with active digital and thumb extension may still increase hand utilization, potentially leading to improved grip strength. This notion aligns with previous findings suggesting a link between hand opening and grip strength (Reinholdt and Fridén, 2013). In this study, the passive opening achieved through certain steps of the grip reconstruction procedure like thumb trapeziometacarpal joint arthrodesis, passive intrinsic reconstruction and EPL tenodesis may facilitate sufficient hand opening, allowing for grip strength comparable with that of the study group. A longer follow-up period may be necessary to fully elucidate the impact of hand opening on grip strength. Therefore, additional studies are warranted to further explore the relationship between hand opening and grip strength.

Reinnervation of active digital extensors after the S-PIN procedure requires a modified approach to grip reconstruction, typically involving less extensive surgical intervention. If active thumb stabilizers (APL, EPL, and EPB) are restored by the S-PIN, then trapeziometacarpal joint arthrodesis may not be necessary; instead, capsulodesis is frequently performed alongside the transfer of the EDM/or EIP to the APB. The EDM to APB transfer was used selectively in patients to improve stability, palmar abduction and thumb pronation during grasp. Full active extension of the little finger (when using the EDM) was not a prerequisite for this transfer, as the APB requires minimal or no force for postural support. In the absence of active function, it may serve as a tenodesis effect.

The patients in the study group did not receive structured rehabilitation following the nerve transfer procedure. Instead, both groups adhered to the tendon transfer rehabilitation protocol (Wangdell et al., 2016), with minimal modifications for those who achieved successful reinnervation after the S-PIN procedure. The potential impact of a tailored rehabilitation protocol specific to the nerve transfer intervention on the outcomes remains to be investigated.

To capture gains in hand opening capacity, the cylinder test served as the primary outcome in this study. This assessment tool provides a standardized and objective method for evaluating grasp and release functions in individuals with neurological injuries. We believe that this measure effectively captures non-functional pinch opening scenarios, such as when the thumb IP or MP joint is hyperflexed or when the thumb is held in rotation. In such instances, measuring the first open web space can be challenging and less meaningful. Additionally, the first web space measurement does not differentiate between radial and palmar abduction of the thumb, which is why we included functional pinch opening, assessed by the maximum active distance between the PIP joint of the index finger and the thumb. The cylinder test is subjected to psychometric evaluation, and preliminary data collected within the scope of this study indicate moderate test–retest reliability. A larger study assessing the psychometric properties of the test is currently underway.

There was no significant difference in activity performance between the groups as measured by the TUAQ. This may be due to the fact that the TUAQ evaluates perceived performance and satisfaction with 10 standardized activities that requires adaptive skill performance. As a result, it may not be sensitive enough to detect changes specifically related to hand-opening ability. However, when comparing the TUAQ scores with those of a patient group from a previous study who had not undergone grasp reconstruction, both performance and satisfaction scores in the previous study were markedly lower. In that study, the mean TUAQ performance interval score was 36.6 (SD = 13.9) out of 100, and the mean satisfaction interval score was 27.5 (SD = 12.1) out of 100 (Wangdell et al., 2022b). In contrast, the groups in the present study had performance scores of 67.3 and 51.8, and satisfaction scores of 50.4 and 40.4, respectively. The earlier cohort provides a natural baseline for what performance and satisfaction typically look like in patients without grasp reconstruction. The consistently higher TUAQ scores in our study suggest that reconstructive surgery contributes to clinically relevant gains, even though direct causal inference cannot be drawn across different study populations.

With the current protocol using a two-stage procedure, incorporating a nerve transfer may delay tendon transfer surgery, as time is needed for reinnervation of the extensor muscles. However, this does not necessarily postpone overall functional recovery. For optimal outcomes, nerve transfers are performed as early as possible, often during the transition from rehabilitation to home, since they are less invasive and require minimal restrictions. This timing allows reinnervation to occur while the patient adapts to new life circumstances, and in most cases, tendon transfer is not delayed but instead aligns naturally with the rehabilitation process. In contrast, tendon transfers place considerably greater demands on the patient in terms of rehabilitation and motivation, which is why they are usually postponed until the patient is fully prepared. In the two-stage protocol, tendon transfer is typically performed about one year after nerve transfer, once reinnervation can be assessed and surgical planning individualized. With increasing knowledge of S-PIN reinnervation, single-stage procedures may now be considered for selected patients. Although simultaneous nerve and tendon transfers could shorten overall rehabilitation time, further studies are needed to determine whether this approach compromises functional outcomes compared with the staged method.

There are limitations in this study. Firstly, the small sample size may have affected the results and limited the generalizability of the findings. Secondly, the outcomes associated with the CNaTT procedure were compared retrospectively to a control group, which may lead to issues with comparability between the groups. However, to minimize variability owing to extraneous variables, we matched the groups on key factors and controlled for covariates that could potentially influence the outcomes. Additionally, the observational design was chosen because its advantages in indicating the potential effects of combining nerve and tendon transfers in tetraplegia outweighed its limitations. This study represents a critical step in providing effect size information for a larger follow-up study.

In conclusion, adding a S-PIN nerve transfer appears to enhance grasp function in the tetraplegic limb when combined with established tendon transfer techniques. While caution is necessary in interpreting the results of this pilot study, the findings are encouraging. To fully demonstrate the superiority of the CNaTT procedure over traditional tendon transfer techniques and to incoporate it in routine practice, more additional participants with a longer follow-up is warranted.
